# A Narrative Review: Analysis of Supplemental Parenteral Nutrition in Adults at the End of Life

**DOI:** 10.3390/ph17010065

**Published:** 2023-12-30

**Authors:** Francisco Rivas García, Rafael Jesús Giménez Martínez, Felipe José Huertas Camarasa, Joan Carles March Cerdá, Fuensanta Lloris Messeguer, Margarita López-Viota Gallardo

**Affiliations:** 1 Municipal Health and Consumer Unit, Guadix City Council, 18500 Guadix, Spain; 2 Department of Nutrition and Bromatology, School of Pharmacy, University of Granada, 18071 Granada, Spain; rafaelg@ugr.es; 3 Chemical Sciences, University of Granada, 18071 Granada, Spain; felipehc@ugr.es; 4 Andalusian School of Public Health, 18011 Granada, Spain; joancarles.march.easp@juntadeandalucia.es; 5 Chemistry Department, IES Zaidín Vergeles, 18008 Granada, Spain; fuensantalloris@ieszaidinvergeles.org; 6 Department of Pharmacy and Pharmaceutical Technology, School of Pharmacy, University of Granada, 18071 Granada, Spain; mlvg@ugr.es

**Keywords:** parenteral nutrition, critical illness, terminally ill, parenteral nutrition solutions, supplemental parenteral nutrition, clinical nutrition, nutritional support

## Abstract

“End of life” is a stage defined by the existence of an irreversible prognosis that ends with a person’s death. One of the aspects of interest regarding end of life focuses on parenteral nutrition, which is usually administered in order to avoid malnutrition and associated complications. However, parenteral nutrition can be adapted to specific circumstances and evolve in its functionality through supplementation with certain nutrients that can have a beneficial effect. This narrative review aims to carry out a situation analysis of the role that could be adopted by supplemental parenteral nutrition in attenuating alterations typical of end of life and potential improvement in quality of life.

## 1. Introduction

“End of life” (EL) is a process that will lead to death due to an illness and/or clinical circumstance with no possible therapeutic alternative. The approach to EL must guarantee physical and mental well-being, which palliative care offers, to reduce the symptoms caused by malnutrition such as edema, nausea, vomiting and reduced appetite. Parenteral nutrition (PN) must be included within the framework of such care as a support whereby hydration and nutrition are guaranteed in EL. PN has been characterized by a rapid technology and formulation evolution since its origin in 1968 in order to cover basic daily nutritional requirements [1,2]. PN is a nutritional strategy that consists of the administration of the nutrients required by the body through the circulatory system. Indications for use of PN include paralytic ileus, intestinal obstruction, short bowel syndrome, acute pancreatitis, intestinal fistulas, very severe previous malnutrition, ulcerative colitis, Crohn’s disease, severe preoperative malnutrition and major surgery. It is contraindicated when the expected intake is less than 5 days, when there is a functioning digestive system and a prognosis that will not improve with PN, and in uncontrolled metabolic alterations. The ultimate objective of PN is the maintenance of an adequate nutritional state, so all the necessary nutrients must be provided to the body in the appropriate proportion [3]. Depending on their nutritional contribution, these nutrients are classified into three large groups as caloric source, protein source and micronutrients. The first group is made up of carbohydrates and fats; the second group is administered through mixtures of amino acids and is quantified in grams of nitrogen; the third group is made up of minerals, trace elements and vitamins. Notwithstanding, PN involves a high financial cost and many complications, as follows.

(A) Thrombotic complications: frequent in 50% of cases and arising from the catheter used, the duration of the procedure, the puncture site and/or the composition of the mixture.
(B) Infectious complications: generated by contamination of the solution, infection of the catheter insertion point, primary contamination and secondary contamination of the catheter.
(C) Metabolic complications: these are diverse and include hyperglycemia, hypoglycemia, hypertriglyceridemia, liver complications, dyslipidemia, bone problems, refeeding syndrome, hypophosphatemia, fat overload syndrome, essential fatty acid deficiency, hyperammonemia and metabolic acidosis [4,5].

The use of PN in EL must be assessed on a case-by-case basis for its impact on the quality of life of the patient. Even though there is controversy about the use of PN in EL since there are positions that justify its use and others that propose its withdrawal or non-establishment because it generates more complications than benefits, there is not enough evidence of the benefits, and the existing benefits are questionable and relate to earlier stages of EL [6]. Notwithstanding, supplemental parenteral nutrition (SPN) represents an innovation, with nutritional supplements assuming increasingly greater physiological relevance. Hence, it is necessary to carry out adequate monitoring of EL with PN to determine the significance of adding nutritional supplements that—due to their beneficial effects—can contribute to improving EL.

## 2. Methodology

This narrative review was conducted through a search for articles on scientific databases. To fully research the importance of supplemental parenteral nutrition at the end of life, a comprehensive study was conducted by searching for studies in Medline, Scopus, Embase and the Cochrane Library.

The following MeSH subject headings were used: “Parenteral Nutrition” (MeSH), “Critical illness” (MeSH), “End of life” (MeSH), “Terminally ill” (MeSH), “Dietary Supplements” (MeSH), “Micronutrients” (MeSH), “Trace elements” (MeSH), “Immunomodulation” (DeSH), “Glutamine” (MeSH), “Oligopeptides” (MeSH), “Drug stability” (MeSH), “Pharmaceutical preparations” (MeSH).

To be as thorough as possible, the appropriate Boolean operators were used, with the search strategy defined by the following:

(parenteral nutrition) AND (critical illness); (parenteral nutrition) AND (end of life); (parenteral nutrition) AND (terminally ill); (parenteral nutrition) AND (dietary supplements); (parenteral nutrition) AND (micronutrients); (parenteral nutrition) AND (trace elements); (parenteral nutrition) AND (immunomodulation); (parenteral nutrition) AND (glutamine); (parenteral nutrition) AND (oligopeptides); (parenteral nutrition) AND (drug stability); (parenteral nutrition) AND (pharmaceutical preparations). Additional studies were searched using references in articles retrieved from the searches.

Final selection of the documents was made in accordance with the established inclusion and exclusion criteria:

− Inclusion criteria: only articles published in indexed journals that included a peer review process were accepted. The inclusion criteria comprised: (a) publications between 1 January 2016 and 1 September 2023; (b) publications on PN supplementation aimed at EL and critical condition; (c) articles written in English and Spanish.
− Exclusion criteria: (a) articles written in languages other than Spanish or English; (b) articles that do not address the human species; (c) articles related to home parenteral nutrition; (d) articles that address pediatric parenteral nutrition; (e) duplicate articles.

Two authors (F-RG and FJ-HC) independently identified the studies and performed data extraction. The percentage of agreement was used to guarantee agreement between evaluators: the number of times that the two reviewers who carried out the evaluation agreed on the same question was added up, and then this figure was divided by the total number of data items considered. To ensure reproducibility and minimize bias, disagreements were resolved through discussion with a third researcher (M-LV).

Initially, 3558 titles and abstracts collected from the different databases consulted were examined. Subsequently, repeated articles were discarded, and the exclusion criteria were applied, resulting in a total of 105 articles taken into account (randomized, observational, cross-sectional, case studies) to be included in the review.

## 3. Impact of Disease and Treatments on Nutritional Status at the End of Life

### 3.1. Nutritional Status and EL

EL is considered a critical status where nutritional support must be aimed at redirecting the metabolic state in order to avoid malnutrition that affects physical health, reduces quality of life and increases morbidity and mortality [7].

The malnutrition accompanying EL occurs mainly with loss of weight and muscle mass, i.e., sarcopenia, with or without loss of fat mass. Malnutrition and weight loss can lead to cachexia, which is a negative predictor of morbidity and mortality in terms of lower tolerance of treatments, higher rate of toxicity and prevalence of infections, worse quality of life and longer duration of hospitalization with increased financial costs [8,9]. Nevertheless, the overall malnutrition status causes a reduction in the capacity to mobilize bronchial secretions and intestinal flora, as well as interstitial edema, atrophy of epithelial cells of the digestive system, decreased ventilatory drive or damage of the cardiac muscles, delay in wound healing, or impairment of cellular and humoral immunity [10,11,12,13].

EL involves an increase in catabolic hormones (catecholamines, corticosteroids and glucagon), which—together with other humoral mediators (IL-1, IL-6, IL-8, TNF-α)—cause alterations in carbohydrate, lipid, and protein metabolism that in turn entails, among other metabolic effects, hypermetabolism, mobilization of energy substrates, blockage of hepatic ketogenesis, and hypercatabolism [14,15].

Hypercatabolism caused by cachexia derives mainly from a systematic inflammatory response that increases the body’s metabolic needs, decreases appetite, and drives catabolism of muscle proteins. Thus, patients with malnutrition exhibit an increase in inflammatory markers (IL6, CRP, β2 microglobulin) due to the presence of an inflammatory state that is associated with malnutrition and cachexia [16].

During EL, a metabolic response to stress is developed aiming to produce the energy required to sustain vital functions, inflammatory response, immune function and tissue repair. Two phases can be distinguished during metabolic stress: hypometabolism and the hypermetabolism phase, which temporally follows the earlier phase [17].

Below are the main metabolic characteristics of EL and their relationship with nutritional status. This outline assumed that EL corresponds to a critical state as addressed by the existing scientific literature.

(a) Carbohydrate Metabolism. Several events to be considered take place, as follows.
  1. *High catabolism without an excessive increase in energy expenditure.* There is an increase in hepatic gluconeogenesis (the substrates used in gluconeogenesis include lactate, alanine and glycerol) and peripheral resistance to the action of insulin, despite the existence of high levels of circulating insulin. These metabolic alterations result in hyperglycemia, which inhibits hepatic glucose production and stimulates its peripheral uptake in order to reduce blood glucose levels [18].
    The high amount of gluconeogenic substrates and the increase in counterregulatory hormones (epinephrine, cortisol and insulin), together with the action of inflammatory mediators, are determining factors for the increase in hepatic glucose production [18,19].
  2. *Inflammatory mediators (TNF-α and interleukins) antagonize the action of insulin and produce hyperglycemia.* Hyperglycemia is common in critically ill patients, even in those patients who have not previously been diagnosed with diabetes. The development of hyperglycemia during EL increases morbidity and mortality, as well as hospital stay days and mechanical ventilation days [20].
  3. *Hyperlactatemia.* Pyruvate and lactate plasma levels are very high during EL. This phenomenon results in a greater conversion of pyruvate to lactate and is more intense at the muscle than at the hepatic level. In this sense, severe hyperlactatemia is associated with extremely high mortality in the intensive care unit (ICU), hence the importance of knowing the variables derived from lactate (timing and persistence of severe hyperlactatemia, maximum level and 12 h clearance) that are associated with mortality [21].
(b) Protein metabolism

Protein metabolism is quite complex in these circumstances, since on the one hand, there is an increase in protein catabolism, and on the other, a decrease in protein synthesis, both in total and visceral proteins. However, hepatic uptake of amino acids and protein synthesis is increased. Numerous events take place and include the following.

1. *Activation of gluconeogenesis to mobilize proteins.* There is a massive mobilization of body nitrogen and an increase in protein catabolism, which is evidenced by increased excretion of urinary nitrogen [22].
2. *Hypoalbuminemia.* The majority of critically ill patients exhibit an inflammatory response that causes endothelial damage and increased capillary permeability, with the ensuing extravasation of fluids and albumin. Hypoalbuminemia represents a marker of increased vascular permeability rather than a marker of albumin itself that is associated with the appearance of edema. In EL, hepatic albumin synthesis would decrease, and both TNF and IL-6 would contribute to this process. Hypoalbuminemia can lead to unnecessary administration of albumin or excessive administration of macronutrients in nutritional regimens, generating possible adverse effects and additional costs, and hence the importance of adequate assessment [23].
3. Excessive catabolism of body proteins that affect skeletal muscle, visceral proteins of connective tissue and circulating proteins. Nutritional support is essential in critically ill patients, and thus great caution must be exercised during the catabolic phase. The use of hypercaloric and hyperprotein nutrition is justified in situations involving marasmus, COPD, respiratory distress syndrome, sepsis with hemodynamic instability, hypercapnia, hyperglycemia and hypertriglyceridemia [24].
4. *Reduction in amino acid metabolism.* It is necessary for nutritional support to maintain adequate metabolic pathways without causing redistributions that compromise the functionality and structure of the organs. The importance of protein intake has been observed in EL patients, resulting in reduction in infectious complications, hospital stay, morbidity associated with energy excess, and in turn reduced mortality in cases of sepsis [25].

(c) Lipid metabolism

Modifications in lipid metabolism are related to the metabolic stress phase. The increase in lipolysis and mobilization of fats causes triglycerides to be released, which are hydrolyzed to free fatty acids and glycerol. Free fatty acids can follow different metabolic pathways, such as: (a) oxidation in skeletal muscle; (b) oxidation in the liver, promoting gluconeogenesis by providing energy and cofactors necessary for glucose synthesis; (c) reesterification in the liver towards triglycerides, resulting in an increase in triglycerides plasma levels. In the event of carnitine deficiency, either prior or acquired, this condition can worsen. Hyperinsulinism, where insulin decreases hepatic ketone production, increases peripheral utilization of fatty acids. This is compounded with a deficiency of long-chain fatty acids, which can lead to an increase in the metabolism of arachidonic acid and appearance of its metabolites with inflammatory properties (prostaglandins and thromboxanes) [26,27,28].

Taking into consideration EL, nutritional support would attenuate metabolic stress, reduce the inflammatory response and avoid malnutrition. Indeed, the use of supplements with immunomodulatory function (Glu, Arg, nucleotides, w-3 and its metabolites and L-carnitine) has shown effectiveness in reducing oxidative damage and a decrease in the metabolic response to stress, although further studies are needed since there are no conclusive data on a reduction in mortality and prevention of PN complications [29,30,31].

### 3.2. Interaction between Nutritional Status and Drugs in PN

As various studies have shown, the treatment of EL causes a deterioration in nutritional status with a decrease in appetite and weight (topiramate, zonisamide, zidovudine, bupropion, fluoxetine, paroxetine, digoxin and digitoxin), as well as alteration of the intestinal microbiota (metformin, brivudine, levodopa and proton-pump inhibitors) [32,33] (Table 1). Therefore, malnutrition can be present in PN if the formula requirements are not administered correctly [7]. Further, some medications have been found to affect micronutrients with diuretics and steroids affecting the absorption of sodium and potassium, while amphotericin B and calcium affect the absorption of magnesium and phosphorus, respectively [34].

It is also worth noting that nutritional status can be affected by pharmacokinetic interactions between nutrients and drugs, which can affect the bioavailability of the nutrient, as well as reduction in the therapeutic action of the drug [32,33].

Pharmacokinetic interactions between drugs and nutrients in PN are the most significant and are limited to the distribution, metabolism and excretion processes of drugs [34]. Thus, an effect of nutrients on the distribution of L-dopa, melphalan, valproic acid, phenytoin, tetracyclines, midazolam, gentamicin and amikacin has been observed, while methotrexate, fluorouracil, lidocaine and theophylline interact during the nutrient metabolism phase [32] (Table 2). Genesis of metabolites from metabolism is to be noted, such as oxalate from the metabolism of ascorbic acid that can interact with calcium and generate insoluble precipitates [34]. A widely debated aspect in the field of nutrient–drug interactions is the influence of amino acid intake on kidney function. Thus, proteins in combination with certain vitamins would affect the metabolism of drugs, causing a delay in their excretion [34].

Even though the addition of drugs in PN is not recommended, for any such addition to take place, absence of risk to the preparation such as degradation and/or destabilization of the lipid emulsion is to be ruled out. It should be noted that combinations of two or more drugs should not be administered in a PN bag and so Y-administration is used, which is nonetheless not free of interactions either. Notwithstanding, there are studies and clinical practice guidelines that have described possible interactions with PN in order to provide recommendations to optimize the PN that is administered [33,34,35].

Therefore, for adequate assessment of the stability and compatibility of the PN concerned with the therapeutic treatment, it is necessary to consider the concentration of amino acids, the type of fat that is part of the parenteral emulsion, the concentration of electrolytes, the concentration of amino acids and dextrose, trace elements, pH, Pka and the acid–base nature of the drugs. In this regard, there are numerous known physical interactions vis-à-vis interactions of a chemical nature that must be further explored and researched. The American Society for Parenteral and Enteral Nutrition (ASPEN) recommends consultation of existing data on physicochemical compatibility and aspects related to therapeutic action prior to preparation of PN [33,34,36].

How can interactions that could worsen the state of malnutrition in EL and/or decrease the therapeutic action of drugs be avoided? The following course of action is proposed.

- Risk assessment through knowledge of all the drugs that the patient is given.
- Monitoring of pharmacological treatment.
- Avoid inappropriate PN mixtures, which will require following the relevant clinical guidelines to avoid PN safety issues.
- Assess the treatment and establish appropriate changes. Establish possible changes in treatment to avoid known interactions that may arise.
- Adapt PN to the administration of drugs according to the patient’s needs.

Malnutrition derived from EL and therapeutic treatment has direct effects on clinical outcomes and is associated with significant spending on medical care, so better identification and treatment of the factors involved in malnutrition related to EL would entail a benefit for people and a potential reduction in health costs [33,34].

## 4. The Role of Parenteral Nutrition at the End of Life: Benefit or Harm?

Nutritional support is an essential part of patient care in EL when oral food intake is not possible. This support includes enteral nutrition (EN) and PN, the use of which will depend on the clinical indications in accordance with the patient’s condition.

Hydration and nutrition are culturally understood as symbols of care for life. Anthropologically, food is related to a basic vital need of people since birth, and it is attributed a meaning of respect for life and care for our fellow human beings. There are positions in favor of considering PN basic care that cannot be waived, and other positions that define it as a basic treatment that can in fact be waived [35,36].

**Figure 1:**
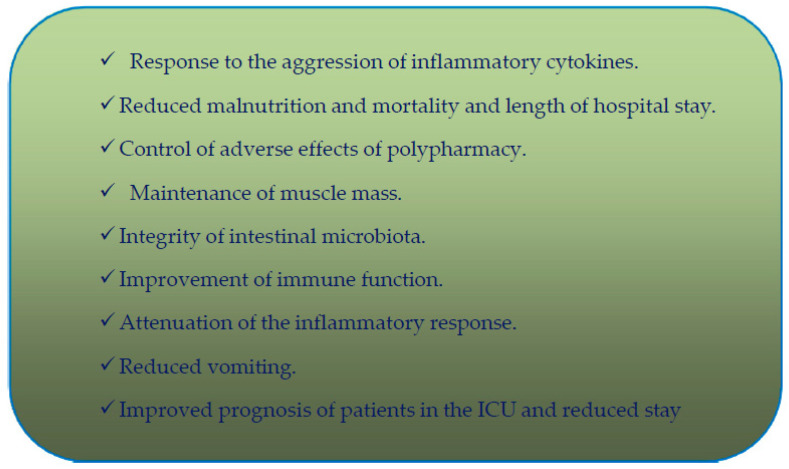
Benefits associated with an adequate nutritional status in EL.

Scientific literature describes a series of benefits associated with an adequate nutritional status in EL (Figure 1): (a) modification of the body’s response to the aggression of inflammatory cytokines; (b) reduced malnutrition associated with hypermetabolism; (c) reduced mortality and length of hospital stay; (d) control of adverse effects of polypharmacy; (e) maintenance of muscle mass; (f) preservation of the integrity of intestinal microbiota; (g) improvement in immune function; (h) attenuation of the inflammatory response; (i) reduced vomiting; (j) improved prognosis of patients in the ICU and reduced stay [37,38,39,40]. However, other studies attribute PN a symbolic value and no significant differences with other life support techniques, which, like any other technique, can sometimes be harmful, since they are considered invasive measures that prolong agony, cause refeeding syndrome, do not reduce mortality or shorten the length of hospital stay and give rise to accumulation of fluids such as bronchial secretions, pleural effusion, infections, edema and ascites [41,42,43,44].

Given these conflicting positions on PN, as well as its imprecise and undefined action protocol under clinical practice guidelines, both the ASPEN and the European Society of Parenteral and Enteral Nutrition (ESPEN) recommend withdrawing and/or not instituting PN if the results are doubtful and do not produce any physical and/or psychological benefit, there is a neurodegenerative pathology, life prognosis is less than 3 days, or for uncontrolled refractory symptoms and multiple organ failure [45,46]. However, it is necessary to continue delving into this field since there are significant gaps to fill and regulate in procedures beyond the individualized approach to each case.

## 5. Supplements of Interest for Parenteral Nutrition Formulations

Nutritional requirements of PN in EL must be established according to the clinical situation, nutritional status, symptomatology of EL, existence of organ failure and the state of hypermetabolism. Therefore, in general, PN will include the following.

- *Carbohydrates.* Carbohydrates constitute the main source of energy. An intake of 4–5 g/kg weight/day is required. Any of the following can be used: (a) glucose solutions that determine osmolarity of the solution and can be used in anhydrous or monohydrate glucose format. Glucose is the preferred carbohydrate due to its usefulness and low cost. It is necessary to ensure that osmolarity does not exceed 1000 Osm/L; (b) glycerol solutions are used mainly in situations where a lower insulin response is desired or there is hyperglycemia due to stress that is difficult to control and thus a glucose substitute is needed. It should be monitored in lipid emulsions that use glycerol since an excess in the formulation can give rise to hemolysis issues; (c) polyols are the least used due to the controversies generated by possible mixture with glucose, fructose, xylitol, sorbitol or glycerol and the relationship with lactic acidosis processes [47].
- *Amino acids.* Essential and non-essential amino acids are used in the form of commercial crystalline amino acid solutions. Daily protein requirements will depend on the degree of metabolic stress and may range between 1 and 2 g/kg weight/day. As to the administration of amino acids, some are usually used as precursors to increase solubility in the parenteral solution, such as tyrosine and cysteine, and others are administered as dipeptides (glutamine, tyrosine) to improve stability and solubility of the formula. Use of branched-chain amino acids in PN has been noted to give rise to controversy since there is lack of consensus on their use as an energy substrate, as well as to increase protein synthesis and turnover. Notwithstanding, in severe cases, the amino acids administered must be modified to adequately control those aspects that may cause an increase in aromatic Aa and alteration of mental status [48].
- *Lipids.* Lipids are responsible for the reduction in osmolarity of the mixture. The contribution of lipids in the form of an O/W emulsion has an energy supply function and a potential positive modulation of the inflammatory response, which is why 1.5–2.5 g/kg weight/day is required. Lipids are incorporated into PN through the administration of essential fatty acids in the form of triglycerides. Fatty acids can be either long chain (from soybean, safflower or sunflower oil), which tend to be more unstable and easily subject to lipid peroxidation, or 50% long- and short-chain mixtures that have shown to be more stable, with fewer liver complications and better nitrogen balance. Also, it has been observed that in these emulsions, tocopherols are transported with fats (affording protection against cellular oxidative damage) and vitamin K (compatible with olive or soy oil). Lipids are used in the form of a lipid emulsion, and emulsifiers (egg lecithin), isotonizers (glycerol) and stabilizers (sodium oleate) are used for stabilization so that the lipid droplets formed are similar to human chylomicrons, but with different types of fatty acids, less cholesterol, no apoproteins and more phospholipids. Intralipid© (soybean oil), Nutralipid© (80% olive oil and 20% soybean oil), Clinolipid© (80% olive oil, 20% soybean oil), SOLE© (soybean oil), SMO Lipid© (olive oil, fish oil, soybean and medium-chain triglycerides) and Omegaven© (fish oil) are among the most common commercial lipid emulsions. In critical situations, different effects in reduction of sepsis and the ratio of T helper and T suppressor lymphocytes have been observed for SOLE©, as well as in the increase in albumin, eicosapentaenoic acid (EPA) and docosahexaenoic acid (DHA) for mixtures of long/short-chain triglycerides [1,49].
- *Electrolytes.* Basal parenteral electrolyte requirements are 1–2 mEq/kg/day of sodium and potassium, 10–15 mEq/day of calcium, 8–20 mEq/day of magnesium, 20–40 mmol/day of phosphate and chlorine and acetate necessary to maintain an acid–base balance. However, the particular EL situation must be assessed, as well as losses that may occur, in order to determine specific daily requirements. Isolated electrolyte preparations comprising sodium, potassium, calcium, chloride, magnesium and phosphorus are used [50].
- *Vitamins.* Fat-soluble vitamins (A, D, E, K) and water-soluble vitamins (B_1,_ B_2,_ B_6,_ B_9,_ B_12_) are included. It is common to have to add supplements based on the recommended needs and requirements of each terminally ill patient [47].
- *Trace elements.* This group includes copper, cobalt, chromium, fluorine, iodine, manganese, molybdenum, nickel, selenium and zinc. Trace element deficiency is associated with various functional and structural abnormalities as they play an important role as enzymatic cofactors. Currently, it is not possible to individualize the prescription of trace elements, but preparations of trace elements already exist [47].
- *Drugs.* The addition of drugs to PN should be avoided whenever possible. However, there are cases in which such addition can be very useful, but drugs can only be added to the bag as long as they do not degrade or destabilize the lipid emulsion and the pharmacokinetics are adequate during administration. In this sense, some of the most commonly used drugs in PN include the following: (a) insulin, which may be necessary in situations of hyperglycemia in patients with preexisting diabetes or in hyperglycemia due to stress; (b) H_2_ antihistamines (ranitidine and famotidine), which tend to be more stable if the PN includes lipids; (c) octreotide and somatostatin, with stability reduced to 24–48 h in the parenteral formulation bag and longer periods leading to adherence to the bag and, consequently, decreased bioavailability; (d) heparin, the addition of which to PN generates controversy since—even though the risk of thrombophlebitis is reduced in people undergoing PN—the interaction that can develop between the negative and positive charges of heparin and calcium from the fat droplets can lead to destabilization and separation of the phases of the emulsion that constitutes the PN, and this needs to be monitored. The fact that the administration of two or more drugs with PN should be avoided should not be ignored [51].
- *Water.* Sterile water is used for injectables, adding just enough to obtain the right volume of the final mixture. A daily amount of water of 30–40 mL/kg weight/day is required [47].

However, SPN involves the addition of nutrients, in standard or individualized formulations, according to the needs of patients and is defined as nutritional support that can be administered when, after individualized patient assessment, the energy and protein intake cannot be covered by PN to guarantee daily requirements [52].

Thus, in EL, SPN would be useful when, after monitoring and assessing the patient, the following is determined: (a) objectives are not achieved with PN and there are increased nutrient requirements; (b) there are nutrient losses; (c) problems arise in absorption and use of nutrients, together with insufficient caloric protein intake; (d) drug–nutrient interactions; (e) hemodynamic instability; (f) hypermetabolism; (g) prolonged use of vasopressor and inotropic medication; (h) intestinal ischemia and high abdominal pressure; and (i) malabsorption. Notwithstanding, SPN must be monitored, as it can cause hyperglycemia, liver dysfunction, and respiratory problems that require the use of mechanical ventilation. It should be noted that SPN is not recommended in the 48 h preceding death [53].

At present, there are no conclusive studies on the use of SPN and its usefulness in EL. Certain studies associate SPN with a decrease in mortality and the risk of infections, while others show that SPN does not reduce the length of stay in the ICU or provide benefits in people who have a low risk of malnutrition [54]. The following are the different nutrients that can make up SPN, as described by scientific literature.

### 5.1. Glutamine

In critically ill patients, glutamine administered as dipeptides of L-alanyl-L-glutamine and glycyl-L-glutamine acts as an essential energy substrate for gluconeogenesis, protein synthesis and as a cellular antioxidant useful for the immune system cells. Critical situations cause a decrease in glutamine levels in the body, which is associated with mortality. Although it can be synthesized by the body, administration of glutamine is necessary since it fulfills a function as a tissue protector, regulator of immune and inflammatory function, attenuating the antioxidant action and protecting metabolism in situations of metabolic stress [55].

At present, there are studies that show its usefulness in critically ill patients after surgery, reducing incidents associated with pneumonia due to mechanical ventilation, as well as reducing the production of cytokines. Also, its use has been associated with a decrease in in-hospital mortality and nosocomial infections [56,57]. However, most recent studies show discrepancies in the use of glutamine, and some of them conclude that although supplementation has been recommended by international societies in their guidelines, there are also data that advise against indiscriminate use in critically ill patients since there is no adequate benefit:risk ratio, in addition to not showing beneficial effects on either the intestinal mucosa or protein metabolism. Supplementation with 0.3–0.5 g/kg/day of glutamine for at least 5 days is recommended, as this may be beneficial in critically ill patients. Therefore, although glutamine administration is recommended in a state of malnutrition, inadequate nitrogen balance and low plasma albumin may ensue [58,59].

### 5.2. Arginine

Arginine is a non-essential amino acid that is nevertheless considered conditionally essential when availability of endogenous arginine is insufficient to meet metabolic demands. Studies that relate arginine with PN in critical conditions have shown that: (a) people postsurgery present more favorable clinical data; (b) there are no immunotoxicity data in formulations with L-arginine; (c) there is safety in administration of arginine by PN; and (d) supplementation reduces mortality and complications due to its ability to stimulate the synthesis of hormones (insulin, glucagon and catecholamines), a useful aspect in the presence of high catabolism. In fact, some immunomodulatory formulas are actually supplemented with this amino acid; arginine is present in concentrations that range from 0.45 g/100 mL to 1.47 g/100 mL. However, it is difficult to obtain conclusive data on the studies carried out with arginine due to the lack of standardization of the different protocols for use of PN, since although an immunomodulatory function is noted, there is uncertainty as to its beneficial role in patients with septicemia [60,61,62,63].

### 5.3. Taurine

Taurine is a non-protein amino acid of great use in patients undergoing home PN, since plasma levels in EL are usually low and so supplementation is highly necessary to cause positive effects on the cardiovascular system, neuroprotection, visual protection and osmoregulation, as well as to exercise antioxidant, anti-inflammatory and immunomodulatory actions. There are no conclusive data regarding the association between taurine and decreased mortality, so despite the immunomodulatory and anti-inflammatory action that taurine could exert, more studies are still needed [64,65].

### 5.4. Citrulline

The interest in citrulline in PN is relatively recent. EL inflammation causes a drop in citrulline. Citrulline is key in the homeostasis of protein synthesis and in nitric oxide metabolism. However, there are currently no clear indications or scientific evidence on its benefit in EL, although there are certain studies that inconclusively relate its presence in PN with a reduction in sepsis and respiratory distress [66].

### 5.5. Lipids

The beneficial role of omega-3 in SPN is observed in the inflammatory response and consequently in the modification of phospholipids of the cell membranes of monocytes, macrophages and other inflammatory cells. Recent studies show that PN supplementation increases survival and produces a reduction in infectious complications, length of hospital stay, duration of mechanical ventilation, and mortality as a result of its anti-inflammatory, antioxidant and protective effect on liver function [67,68,69].

### 5.6. Electrolytes

Electrolytes (chloride, phosphate, acetate, sodium, potassium, calcium and magnesium) play a fundamental role in regulating homeostasis in EL. There is no standard or protocolized pattern in SPN and the benefit/risk will have to be considered based on the patient’s physiological conditions [47]. However, certain considerations regarding SPN electrolytes can be of use: (a) sodium supplementation should be considered with respect to water balance; (b) the rate of perfused potassium delivery should be monitored to avoid potential cardiac arrest; (c) phosphate supplementation needs to be adequate to restore the intracellular phosphate deficit and compensate for the drop in plasma phosphate in EL, which is usually linked to malnutrition, hyperglycemia and/or renal failure; (d) phosphate is available as a sodium or potassium derivative, magnesium is normally supplied as magnesium sulfate, and calcium is available as calcium gluconate [70]. The mode and time of incorporation of electrolytes into the PN is of great importance, since they may be responsible for the stability and safety of the lipid emulsion that forms the SPN.

### 5.7. Trace Elements

Trace elements play an important role in SPN, but should not be used routinely without prior clinical assessment. The risk factors of deficiency of trace elements include malabsorption, chronic kidney or liver pathology and malnutrition, as well as nutrient–drug interactions (isoniazid, phenobarbital, penicillin, theophylline and cyclic antidepressants). However, excess trace elements can be constitutive of toxicity and absence of necessary nutrients that would imply the genesis of various pathologies and physiological alterations.

The main considerations regarding the current situation of trace elements and SPN are described next for the trace elements most commonly used in SPN.

*Zinc.* Zinc deficiency gives rise to an increase in proinflammatory cytokines. Zinc administration in the form of zinc sulfate causes the formation of zinc complexes with aspartate, cysteine, histidine or methionine for increased absorption. Zinc supplementation is not common practice, nor is it included in clinical guidelines except in patients who receive PN without zinc and have high intestinal losses [71].

*Selenium.* The inflammatory process that characterizes EL causes a decrease in selenium concentration and hence a decrease in the body’s antioxidant response capacity. Selenium has been determined to have a beneficial effect on the formation of selenoproteins with antioxidant action against free radicals that are formed as a consequence of the metabolic state in EL [72]. However, there are no conclusive data on its possible action in reducing mortality or the worsening that may occur in patients with kidney failure [73].

*Chromium.* Chromium promotes the action of insulin, improving its activity in peripheral tissue. Therefore, chromium administration causes better glucose control, a useful aspect in hyperglycemia, which is why it is only supplemented with chromium in cases of hyperglycemia and to reduce insulin requirements [74].

*Cobalt.* Cobalt relates to the metabolism and use of vitamin B_12_. Supplementation is not needed if PN is already high in vitamin B_12_ [75].

*Iodine, Manganese, Iron, Molybdenum.* ASPEN guidelines recommend their supplementation given their regulatory functions in metabolism and immunomodulation [75].

*Coenzyme Q.* This can be an interesting supplement that still requires additional studies, but whose synergistic interaction with selenium would show benefits for cellular energy supply, as well as antioxidant properties [22,75,76].

*L-Carnitine.* Supplementation is recommended in the event of hypertriglyceridemia and hyperlactatemia in cases of prolonged PN [75].

*Probiotics.* It has been suggested that SPN with probiotics reduces the need for mechanical ventilation in chronic conditions arising from nosocomial infections. Additional studies are needed for consideration of the use of species of the Lactobacillus, Bifidobacterium and Streptococcus genera [77].

*Nucleotides.* Nucleotides are usually insufficient to cope with the catabolic stress of EL. Although additional studies are required to confirm the scientific evidence regarding their benefits, to date usefulness has been established to prevent deterioration of the intestinal barrier and therefore to reduce infectious complications [78].

*Vitamins.* EL entails vitamin deficiencies that require supplementation, and thus vitamins are administered as a commercial multivitamin supplement composed of vitamins C, A, E, K and the B complex (B_1_, B_2_, B_3_, B_6_, B_7_, B_9_, B_12_) [79]. The literature includes a series of considerations with respect to SPN and vitamins: (a) there is no conclusive evidence of or studies on the possible benefit of vitamin D, although there are studies that associate it with a decrease in mortality and mechanical ventilation time; (b) vitamin C is unstable, susceptible to oxidation reactions and is usually associated with neuropathy, hemolysis and hyperglycemia phenomena [80,81]; (c) B group vitamins are involved in reducing fatigue syndrome [82]; (d) vitamin supplementation is justified in terms of nutritional requirements to improve immune function, regulate hypercatabolism and exert an antioxidant action, but if it is provided too early, it can have adverse effects such as prolonging the ICU stay and the risk of infections [83]; (e) ASPEN and ESPEN clinical practice guidelines refer to the need to supplement PN with vitamins in critical condition, but there is no consensus or evidence on timing, clinical criteria or protocols in EL [84].

*Glutathione.* Glutathione constitutes at present a line of research, currently in animal trials, studying the possible protective effect of glutathione to prevent the effects of PN on hepatic metabolism. Although only animal model results are available, consideration thereof is still important, as perhaps studies in humans can be conducted in the near future [85].

## 6. Compatibility and Stability of Nutrients in Parenteral Nutrition of Interest for SPN

Relevance of compatibility and stability, which are two different aspects in PN, are to be noted. Stability refers to possible physical, chemical or microbiological degradation, while compatibility comprises everything related to loss of the properties of the parenteral formulation, both in terms of nutrients and the possible toxicity that may be generated. Consequently, the requirements of PN stability and compatibility must persist over time from preparation to administration to guarantee its safety and effectiveness [86]. There are diverse factors that affect the compatibility and stability of PN that are also applicable to SPN. Hence, this section addresses general aspects of PN that will also have to be considered in the event that SPN is available, given the fact that to date, the bibliography does not describe any aspects that differ much between PN and SPN with regard to compatibility and stability. However, differentiating nuances that may exist between PN and SPN are also discussed.

### 6.1. Lipid Emulsion

PN lipid emulsions are O/W-type and thermodynamically unstable since the lipid droplets tend to join and break the emulsion. Therefore, stability of the lipid component of SPN will largely determine the stability and safety of the final preparation [86]. In this regard, there are a series of factors that affect stability of the lipid emulsion, as follows.

- Emulsifying agents. They maintain stability due to the establishment of repulsive forces between the phases, which prevent the fusion of fat droplets [87]. The use of emulsifiers such as phospholipids, glycerol and lysoderivatives of lecithin in the emulsion is relevant, as they cause negative repulsive forces on the surface that help maintain lipid stability [88].
- Z potential. It is the parameter that determines stability of the emulsion and size of the particles and should always be maintained above −1.5 mV to avoid irreversible destabilization of the emulsion caused by a decrease in electrostatic repulsion generating flocculation, cremation, coalescence and phase inversion [1].
- Amino acids. Their main function is to prevent instability of emulsions by avoiding the precipitation of calcium and trace elements, enhancing action of emulsifiers and acting as buffers when pH is low. Greater stability is achieved with a ratio of acidic amino acids/base amino acids under 1.5 and pH higher than the isoelectric point of the amino acids [88,89].

Notwithstanding, reactivity of amino acids with the following should be taken into account: (a) glucose, causing the Maillard reaction that results in a reduction in bioavailability of amino acids and loss of lysine, threonine and histidine [90]; (b) vitamins: cysteine promotes oxidation of ascorbic acid, while histidine, methionine and tryptophan can undergo oxidation reactions in the presence of vitamins [90]; (c) trace elements used in SPN binding of zinc with histidine, cysteine, glutamine and taurine have been observed without affecting stability and leading to a positive effect on bioavailability.

pH can also promote glutamine, aspartame, arginine and histidine complexes, which are indirectly involved in calcium phosphate precipitation. However, oxidation of tryptophan, cysteine and histidine occurs in SPN bags that allow oxygen permeability [88].

Therefore, the independent pH of each of the PN components of the mixture must be taken into account, since this determines the final pH and stability. In fact, final pH will be a determining element to prevent calcium phosphate precipitation [90].

- Droplet size. The droplet size should not exceed 6 μm, since this can lead to circulatory problems (embolism and/or thrombosis) and liver and kidney damage. A large droplet deconstructs the emulsion, and therefore anything that conditions and/or increases droplet size affects stability and safety of the emulsion [88,89].
- Type of triglycerides of fatty acids. The lipid destabilization process can produce various phenomena such as creaming, flocculation, and in some cases emulsion breakage. The use of long-chain triglycerides (LCTs), medium-chain triglycerides (MCTs), and polyunsaturated fatty acids (PUFAs), together with phytosterols and α-tocopherols, exerts a protective effect on lipid peroxidation of the emulsion and provides a beneficial element, given their anti-inflammatory effects. In this regard, the presence of MCTs seems to reduce the destabilizing effects of long-chain triglycerides, resulting in more stable emulsions. New lipid emulsions rich in omega-3 fatty acids, MCTs and LCTs are being currently used, which a priori appear to be as stable as MCT–LCT mixtures. The use of emulsifiers such as phospholipids, glycerol and lysoderivatives of lecithin causing negative repulsive forces on the surface that help maintain lipid stability is relevant [88,89,91,92,93,94,95].
- pH. The increase in pH is directly proportional to the stability of the emulsion. Thus, a value of 7–8 would be optimal, while coalescence of the fat droplets would take place when under 5. Low concentration of amino acids, heat sterilization processes and an inadequate base-to-acid amino acid ratio under 1.5 are related to a decrease in pH to values that affect the PN, with precipitation of calcium phosphate, copper-cysteine and folic acid [88,96,97].
- Glucose concentration. This should be properly determined to avoid a low concentration that would entail the risk of calcium phosphate precipitation, or a high concentration that would determine emulsion breakage [88]. Furthermore, an increase in the diameter of the fat droplets is triggered if glucose is added directly to the emulsion, given the acidic pH of glucose solutions and presence of ions that are attached to the fat globules [89].
- Ions and cations. They can affect the stability of the lipid emulsion, which is why the amount of electrolytes should be checked and monitored, specifically di- and trivalent cations. It has been shown that the calcium cation and the acetate anions exert a protective effect on the emulsion by presenting buffer properties, while the di- and trivalent cations are destabilizing. Calcium salts, sodium acetate, magnesium sulfate and calcium gluconate are often used in SPN commercial preparations to ensure stability [1,80,88].
- Lipid peroxidation reaction. Peroxidation or lipoperoxidation reaction is a lipid oxidative degradation reaction. This oxidative lipid degradation reaction occurs in the presence of oxygen and is increased in the presence of ultraviolet radiation. Due to the high number of double bonds, polyunsaturated fatty acids are susceptible to peroxidation. Greater formation of peroxides is observed in emulsions based on soybean oil than in structured lipids, mixtures of MCTs, LCTs and/or in emulsions based on soybean oil [47,88,89,98]. SPN containing low concentrations of alpha-tocopherol generate few peroxides since alpha-tocopherol acts as an antioxidant, although a prooxidant effect has been observed at high concentrations [89,99,100].

### 6.2. Trace Elements

Scientific literature shows that some trace elements used in SPN can generate insoluble salts that precipitate when forming complexes, as is the case with copper, selenium, zinc and manganese. Also, storage and refrigeration can affect the concentration of selenium and manganese [88,101]. Trace elements are involved in lipid flocculation, catalyzing oxidation-reduction reactions, vitamin degradation and formation of complexes with amino acids. Thus, pH can exert an effect since precipitates such as dihydroxides are generated if pH is greater than 5.5. Precipitation of calcium and zinc also takes place through the genesis of insoluble metal sulfides [89,102].

### 6.3. Factors Leading to Precipitate Formation

One of the most relevant aspects affecting stability of PN and SPN formulations is the precipitation of calcium and phosphate, which is determined by: (a) an increase in pH that generates more dibasic phosphate; (b) an increase in temperature that results in dissociation of calcium; (c) calcium, magnesium and phosphate concentrations; (d) a low concentration of amino acids; (e) the order of addition into the mixture; (f) conservation time; (g) administration conditions (perfusion rate and ambient temperature); (h) use of diacid phosphate; (i) an increase in the concentration of divalent calcium; (j) high concentration of amino acids rich in phosphate and gluconate; and (k) low dextrose concentration. Precipitation not only implies a physicochemical alteration, but is also associated with toxicity (respiratory distress, embolism, interstitial pneumonitis) and loss of activity of related drugs. Thus, the presence of divalent and trivalent minerals forms complexes with tetracyclines, ciprofloxacin, and the oxalate from vitamin C can interact with calcium, generating precipitates [20,70,88,89,102]. To prevent formation of these precipitates, action can be taken to control concentrations of calcium and phosphate by reducing the pH, as this would generate dibasic phosphate. The fact that a low concentration of amino acids would enhance the formation of precipitates due to their buffering effect as well as other factors such as the mixing order, storage time, infusion rate and ambient temperature should be taken into account. A low concentration of dextrose also favors the formation of precipitates [20,70,88,89,102].

### 6.4. Factors Affecting Vitamin Stability

Factors that can affect vitamin stability and therefore generate technological problems include photolysis (by ultraviolet and fluorescent light), adsorption in the bag and oxidation-reduction reactions, all accelerated by an increase in temperature [20]. Specifically, inactivation of vitamin C has been observed in the presence of copper and oxygen, while vitamins B_1_, B_9_, B_6_ are inactivated in the presence of iron. It should also be noted that an alkaline pH inactivates water-soluble vitamins and vitamin A. Other interactions have taken place between the combination of vitamin K and B_12_, leading to loss of vitamin B_12_. The role of light and trace elements in degradation of fat-soluble vitamins by photoperoxidation is particularly relevant, given that this gives rise to destruction of vitamins and the genesis of radicals that can worsen the state of EL [20,88,89].

Notwithstanding, certain vitamins deserve special attention in relation to their stability in PN/SPN and the various factors that can affect them as follows.

(a) Vitamin C. It can undergo chelating processes due to cysteine content of the amino acid, as well as oxidative processes due to oxygen permeability of the bag. The degradation processes that can develop due to a temperature increase are of interest [88,89].
(b) Vitamin A. It is degraded by photoperoxidation, which implies the need for photoprotection of the bag. However, there are discrepancies regarding adsorption of said vitamin in the PVC bag, which could influence the rate of intestinal absorption [88,89].
(c) Vitamin E. It presents greater stability when protected from light and refrigerated.
(d) Group B vitamins. Their stability depends on photoprotection, temperature and storage conditions. Special aspects include the fact that increased stability of folic acid is associated with PVC bags and that vitamin B_1_ presents more stability in the absence of bisulfites in the amino acid solution. On the other hand, refrigeration affords greater stability to vitamin B_12_, while the stability of vitamins B_2_ and B_9_ is conditioned by the presence of oxygen and pH [20,88,89].

### 6.5. Mixing Order of the Components

During preparation of PN, lipid emulsions must be added at the last possible moment to obtain the least possible percentage of globules with a size greater than 6 μm and thus avoid the formation of precipitates. Clinical practice guidelines and ASPEN guidelines establish that an adequate mixing order that avoids the formation of precipitates will follow the order of amino acid solutions and phosphate source, glucose solutions, vitamins, sodium and potassium, trace elements, divalent cations, electrolyte solution, lipids, and calcium [90]. This takes on special relevance in SPN, since the supplements must be added at the end of the preparation to avoid precipitation and/or breakage of the emulsion.

### 6.6. Addition of Drugs

Considering the risk of safety and stability of PN after incorporation of drugs in the same bag, ASPEN clinical practice guidelines recommend not making mixtures except in cases in which there is scientific documentation that takes into consideration the concentration of amino acids, glucose, dextrose and electrolytes, the type of emulsion fat, the type and concentration of trace elements, pH and Pka, acidic or base nature of the mixture as a whole, as well as the additives of the drug concerned, and that shows that the mixture will not cause compatibility or stability issues and therefore that the therapeutic action of the treatment will not be compromised [2,20].

### 6.7. Temperature

Refrigeration of PN is the best conservation technique to prolong its useful life when faced with high temperatures that lead to an increase in reactivity and interactions between the components, risk of calcium phosphate and ascorbic acid precipitation, as well as dissociation of calcium salts that generates free calcium ions that can form complexes with phosphate salts [89].

### 6.8. Storage Bags

Oxygen and/or light are factors that negatively affect the stability of PN components, especially of vitamins (used in SPN) and lipids. Oxidation of some vitamins and lipid peroxidation are catalyzed by light, with the presence of oxygen being the decisive factor in the process. Therefore, the material of the bag in contact with the PN must be chemically inert and avoid the presence of components that adsorb other components of the PN, with the most accepted being the EVA (ethylene-vinyl acetate) bag, which has undergone various modifications over time. The modification that stands out the most is the new multilayer EVA bag that provides total non-permeability to oxygen. In addition, the multilayer EVA bag also provides certain photoprotective action against both UVA and UVB radiation [2,20,103].

Three-chamber bags include an electrolyte compartment and are equipped with ports for controlled addition of trace elements, vitamins and micronutrients (vitamins and trace elements) that can be added to the reconstituted mixture as needed. In this regard, EVA bags should prevent oxidation-reduction reactions (oxygen plus high temperature and humidity), hydrolysis (dependent on temperature and pH), and photochemical reactions (conditioned by resistance, porosity and elasticity of the bag). Filters can be used throughout the filling process to prevent particles of variable size from entering the bag and posing a considerable risk, such as catheter obstruction or pulmonary embolism.

After preparation of the PN and until its administration, it should be stored and preserved under certain conditions, such as at a temperature of 4–8 °C and guarded from light to guarantee its stability, compatibility and safety. It is advisable to administer the PN as soon as possible and not allow it to remain for more than 24 h at room temperature [20,103,104,105].

## 7. Conclusions

EL is a disaster drawer with no protocol, recommendations or guidelines defined in clinical practice guidelines that can be standardized and applied to everybody, but rather, the nutritional status and clinical symptoms resulting in EL must be assessed individually. All this is required to ensure the best decisions regarding the establishment of SPN after considering its benefits and disadvantages. At present, there are numerous doubts about the real role that SPN can play, and they have not been resolved or adequately studied, given that some studies show a positive action, others show a negative one, and others do not attribute any type of action to SPN, but all of them agree on the need to continue conducting more research. This article has addressed the usefulness of SPN in EL in terms of justification given the situation of hypermetabolism, the different supplements involved, and the techno-pharmaceutical elements of interest to guarantee safety and stability. All this represents a starting point for a more exhaustive and in-depth line of research that allows us to open up a specific area of study on the different types of supplements used in PN in daily clinical practice and their potential beneficial action to reduce symptomatology in order to help improve the quality of life of patients, as well as to optimize certain health resources that could be achieved with SPN.

## Figures and Tables

**Table 1:** Nutritional status and drugs.

Drugs	Alterations
Topiramate, zonisamide, zidovudine, bupropion, fluoxetine, paroxetine, digoxin, digitoxin	Decrease in appetite and weight
Metformin, brivudine, levodopa and proton-pump inhibitors	Intestinal dysbiosis

**Table 2:** Pharmacokinetic interactions between drugs and nutrients in PN.

Nutrients	Drugs	Pharmacokinetic Phase Interaction
Calcium, magnesium, sodium, vitamins (A, D, E, K).	L-dopa, melphalan, valproic acid, phenytoin, tetracyclines, midazolam, gentamicin, amikacin	Distribution, metabolism, excretion
Vitamin B_12_, calcium, sodium, protein, vitamin D.	Methotrexate, fluorouracil, lidocaine theophylline	Metabolism

## Data Availability

Data sharing not applicable.

## References

[1] Rivas-García F., Giménez-Martínez R., López-Viota Gallardo M. (2022). Nutrición parenteral suplementada en el final de la vida: Consideraciones nutricionales y tecnológicas. Ars Pharm..

[2] Reber E., Messerli M., Stanga Z., Mühlebach S. (2019). Pharmaceutical Aspects of Artificial Nutrition. J. Clin. Med..

[3] (2023). Bioética, Bioderecho y soporte nutricional en el final de la vida. Francisco Rivas García. Madrid, Editorial Universitas. https://universitas.es/product-category/coleccion-bioetica-y-pensamiento-juridico/.

[4] Berlana D. (2022). Parenteral Nutrition Overview. Nutrients.

[5] Rivas-García F., López-Viota-Gallardo M. (2023). Actualización en dispositivos sanitarios para administración de nutrición parenteral. Ars Pharm..

[6] Holdoway A. (2022). Nutrition in palliative care: Issues, perceptions and opportunities to improve care for patients. Br. J. Nurs..

[7] Ciliz O., Tulek Z., Hanagasi H., Bilgic B., Gurvit I.H. (2023). Eating Difficulties and Relationship with Nutritional Status among Patients with Dementia. J. Nurs. Res..

[8] Sambataro D., Politi M.R., Messina A., Scarpello L., Messina S., Guggino R., Carnaghi C., Caccialanza R., Gebbia V. (2023). Relationship of Inflammatory Parameters and Nutritional Status in Cancer Patients. Anticancer Res..

[9] (2017). Bozzetti F: Forcing the vicious circle: Sarcopenia increases toxicity, decreases response to chemotherapy and worsens with chemotherapy. Ann. Oncol..

[10] Tortuyaux R., Davion J.B., Jourdain M. (2022). Intensive care unit-acquired weakness: Questions the clinician should ask. Rev. Neurol..

[11] Mentella M.C., Scaldaferri F., Pizzoferrato M., Gasbarrini A., Miggiano G.A.D. (2020). Nutrition, IBD and Gut Microbiota: A Review. Nutrients.

[12] Cristina N.M., Lucia D. (2021). Nutrition and Healthy Aging: Prevention and Treatment of Gastrointestinal Diseases. Nutrients.

[13] Yamada S., Arase H., Yoshida H., Kitamura H., Tokumoto M., Taniguchi M., Hirakata H., Tsuruya K., Nakano T., Kitazono T. (2022). Malnutrition-Inflammation Complex Syndrome and Bone Fractures and Cardiovascular Disease Events in Patients Undergoing Hemodialysis: The Q-Cohort Study. Kidney Med..

[14] Stolarski A.E., Young L., Weinberg J., Kim J., Lusczek E., Remick D.G., Bistrian B., Burke P. (2022). Early metabolic support for critically ill trauma patients: A prospective randomized controlled trial. J. Trauma Acute Care Surg..

[15] Benjamin A., John S. (2023). Malnutrition and undernutrition: Causes, consequences, assessment and management. Medicine.

[16] Merker M., Felder M., Gueissaz L., Bolliger R., Tribolet P., Kägi-Braun N., Gomes F., Hoess C., Pavlicek V., Bilz S. (2020). Association of baseline inflammation with effectiveness of nutritional support among patients with disease-related malnutrition: A secondary analysis of a randomized clinical trial. JAMA Netw. Open.

[17] Hoffer L.J., Bistrian B.R. (2016). Nutrition in critical illness: A current conundrum. F1000Research.

[18] Marian M. (2015). Carbohydrate Metabolism. A comparison of stress and non stress states. Nutrition for the Critically Il Patient. A Guide to Practice.

[19] Becker C.D., Sabang R.L., Nogueira Cordeiro M.F., Hassan I.F., Goldberg M.D., Scurlock C.S. (2020). Hyperglycemia in Medically Critically Ill Patients: Risk Factors and Clinical Outcomes. Am. J. Med..

[20] Silveira Rossi J.L., Barbalho S.M., Reverete de Araujo R., Bechara M.D., Sloan K.P., Sloan L.A. (2022). Metabolic syndrome and cardiovascular diseases: Going beyond traditional risk factors. Diabetes Metab. Res. Rev..

[21] Gharipour A., Razavi R., Gharipour M., Modarres R., Nezafati P., Mirkheshti N. (2021). The incidence and outcome of severe hyperlactatemia in critically ill patients. Intern. Emerg. Med..

[22] Van Gassel R.J.J., Baggerman M.R., van de Poll M.C.G. (2020). Metabolic aspects of muscle wasting during critical illness. Curr. Opin. Clin. Nutr. Metab. Care.

[23] Erstad B.L. (2021). Serum Albumin Levels: Who Needs Them?. Ann. Pharmacother..

[24] Lambell K.J., King S.J., Forsyth A.K., Tierney A.C. (2018). Association of Energy and Protein Delivery on Skeletal Muscle Mass Changes in Critically Ill Adults: A Systematic Review. J. Parenter. Enter. Nutr..

[25] O’Keefe G.E., Shelton M., Qiu Q., Araujo-Lino J.C. (2019). Increasing Enteral Protein Intake in Critically Ill Trauma and Surgical Patients. Nutr. Clin. Pract..

[26] De Waele E., Malbrain M.L.N.G., Spapen H. (2020). Nutrition in Sepsis: A Bench-to-Bedside Review. Nutrients.

[27] Martin-Perez M., Urdiroz-Urricelqui U., Bigas C., Benitah S.A. (2022). The role of lipids in cancer progression and metastasis. Cell Metab..

[28] Broadfield L.A., Pane A.A., Talebi A., Swinnen J.V., Fendt S.M. (2021). Lipid metabolism in cancer: New perspectives and emerging mechanisms. Dev. Cell.

[29] Malekahmadi M., Pahlavani N., Firouzi S., Clayton Z.S., Islam S.M.S., Rezaei Zonooz S., Moradi Moghaddam O., Soltani S. (2022). Effect of enteral immunomodulatory nutrition formula on mortality and critical care parameters in critically ill patients: A systematic review with meta-analysis. Nurse Crit. Care.

[30] Hirschberger S., Schmid A., Kreth S. (2023). Immunmodulation durch Ernährung bei kritisch kranken Patienten [Immunomodulation by nutritional intervention in critically ill patients]. Anaesthesiologie.

[31] Tejera Pérez C., Guillín Amarelle C., Rodríguez Novo N., Lugo Rodríguez G., Mantiñán Gil B., Palmeiro Carballeira R., Pita Gutiérrez F., Argüeso Armesto R., Cantón Blanco A., Botana López M.A. (2023). Inmunonutrición, evidencias y experiencias [Immunonutrition, evidence and experiences]. Nutr. Hosp..

[32] Fort Casamartina E., Arribas Hortiguela L., Bleda Pérez C., Muñoz Sánchez C., Peiro Martínez I., Perayre Badía M., Clopés Estela A. (2016). Interacción entre tratamientos oncológicos y soporte nutricional. Nutr. Hosp..

[33] Ased S., Wells J., Morrow E.L., Malesker M.A. (2018). Clinically significant food-drug interactions. Consult. Pharm..

[34] Deng J., Zhu X., Chen Z., Fan C.H., Kwan H.S., Wong C.H., Shek K.Y., Zuo Z., Lam T.N. (2017). A review of food-drug interactions on oral drug absorption. Drugs.

[35] Choi J.H., Ko C.M. (2017). Food and drug interactions. J. Lifestyle Med..

[36] Boullata J.I., Carrera A.L., Harvey L., Escuro A.A., Hudson L., Mays A., McGinnis C., Wessel J.J., Bajpai S., Beebe M.L. (2017). ASPEN safe practices for enteral nutrition therapy [formula: See text]. J. Parenter. Enter. Nutr..

[37] Cordeiro L.A.F., Silva T.H., de Oliveira L.C., Neto J.F.N. (2020). Systemic Inflammation and Nutritional Status in Patients on Palliative Cancer Care: A Systematic Review of Observational Studies. Am. J. Hosp. Palliat. Care.

[38] Del Olmo García M.D., Moreno Villares J.M., Álvarez Hernández J., Ferrero López I., Bretón Lesmes I., Virgili Casas N., Ashbaugh Enguídanos R., Lozano Fuster F.M., Wanden-Berghe C., Irles Rocamora J.A. (2022). Nutrición en cuidados paliativos: Resumen de recomendaciones del Grupo de Trabajo de Ética de la SENPE [Nutrition in palliative care: Guidelines from the Working Group on Bioethics, Spanish Society of Clinical Nutrition and Metabolism (SENPE)]. Nutr. Hosp..

[39] Morais S.R. (2016). Nutrition, quality of life and palliative care: Integrative review. Rev. Dor. Sao Paulo.

[40] Marcolini E.G., Putnam A.T., Aydin A. (2018). History and Perspectives on Nutrition and Hydration at the End of Life. Yale J. Biol. Med..

[41] Boulanger A. (2017). Opinions about the new law on end-of-life issues in a sample of french patients receiving palliative care. BMC Palliat. Care.

[42] Cotogni P. (2016). Enteral versus parenteral nutrition in cancer patients: Evidences and controversies. Ann. Palliat. Med..

[43] Carter A.N. (2020). To What Extent Does Clinically Assisted Nutrition and Hydration Have a Role in the Care of Dying People?. J. Palliat. Care.

[44] Ridley E.J., Davies A.R., Parke R., Bailey M., McArthur C., Gillanders L., Cooper D.J., McGuinness S., Supplemental Parenteral Nutrition Clinical Investigators (2018). Supplemental parenteral nutrition versus usual care in critically ill adults: A pilot randomized controlled study. Crit. Care..

[45] Allingstrup M.J., Kondrup J., Wiis J., Claudius C., Pedersen U.G., Hein-Rasmussen R., Bjerregaard M.R., Steensen M., Jensen T.H., Lange T. (2017). Early goal-directed nutrition versus standard of care in adult intensive care patients: The singlecentre, randomised, outcome assessor-blinded EAT-ICU trial. Intensive Care Med..

[46] Druml C., Ballmer P.E., Druml W., Oehmichen F., Shenkin A., Singer P., Soeters P., Weimann A., Bischoff S.C. (2016). ESPEN guideline on ethical aspects of artificial nutrition and hydration. Clin. Nutr..

[47] ASPEN (2020). Parenteral Nutrition Handbook.

[48] Iacone R., Scanzano C., Santarpia L., Cioffi I., Contaldo F., Pasanisi F. (2020). Macronutrients in Parenteral Nutrition: Amino Acids. Nutrients.

[49] Sadu Singh B.K., Narayanan S.S., Khor B.H., Sahathevan S., Gafor A.H.A., Fiaccadori E., Sundram K., Karupaiah T. (2020). Composition and Functionality of Lipid Emulsions in Parenteral Nutrition: Examining Evidence in Clinical Applications. Front. Pharmacol..

[50] Laesser C.I., Cumming P., Reber E., Stanga Z., Muka T., Bally L. (2019). Management of Glucose Control in Noncritically Ill, Hospitalized Patients Receiving Parenteral and/or Enteral Nutrition: A Systematic Review. J. Clin. Med..

[51] McCulloch A., Bansiya V., Woodward J.M. (2018). Addition of Insulin to Parenteral Nutrition for Control of Hyperglycemia. J. Parenter. Enter. Nutr..

[52] Li P., Zhong C., Qiao S., Liu J. (2022). Effect of supplemental parenteral nutrition on all-cause mortality in critically Ill adults: A meta-analysis and subgroup analysis. Front. Nutr..

[53] Singer P. (2019). Preserving the quality of life: Nutrition in the ICU. Crit. Care.

[54] Ridley E.J. (2021). Parenteral nutrition in critical illness: Total, supplemental or never?. Curr. Opin. Clin. Nutr. Metab. Care.

[55] Smedberg M., Wernerman J. (2016). Is the glutamine story over?. Crit. Care..

[56] Sun Y., Zhu S., Li S., Liu H. (2021). Glutamine on critical-ill patients: A systematic review and meta-analysis. Ann. Palliat. Med..

[57] Singer P., Blaser A.R., Berger M.M., Alhazzani W., Calder P.C., Casaer M.P., Hiesmayr M., Mayer K., Montejo J.C., Pichard C. (2019). ESPEN guideline on clinical nutrition in the intensive care unit. Clin. Nutr..

[58] Cruzat V., Macedo Rogero M., Noel Keane K., Curi R., Newsholme P. (2018). Glutamine: Metabolism and Immune Function, Supplementation and Clinical Translation. Nutrients.

[59] van Zanten A.R.H., De Waele E., Wischmeyer P.E. (2019). Nutrition therapy and critical illness: Practical guidance for the ICU, post-ICU, and long-term convalescence phases. Crit. Care..

[60] Rosenthal M.D., Carrott P.W., Patel J., Kiraly L., Martindale R.G. (2016). Parenteral or Enteral Arginine Supplementation Safety and Efficacy. J. Nutr..

[61] Ginguay A., De Bandt J.P., Cynober L. (2016). Indications and contraindications for infusing specific amino acids (leucine, glutamine, arginine, citrulline, and taurine) in critical illness. Curr. Opin. Clin. Nutr. Metab. Care.

[62] Gupta A., Gupta E., Hilsden R., Hawel J.D., Elnahas A.I., Schlachta C.M., Alkhamesi N.A. (2021). Preoperative malnutrition in patients with colorectal cancer. Can. J. Surg..

[63] Al-Toubah T., Sikaria D., Jesurajan J., Bottiglieri S., Smith J., Pellé E., Hutchinson T., Strosberg J., EL-Haddad G. (2021). Comparison of Nausea and Vomiting Associated with Amino Acid Formulations Coinfused with Peptide Receptor Radionuclide Therapy: Commercial Parenteral Nutrition Formulas versus Compounded Arginine/Lysine. Pancreas.

[64] Korzilius J.W., Gillis V.E.L.M., Wouters Y., Wanten G.J.A. (2022). Taurolidine-related adverse events in patients on home parenteral nutrition frequently indicate catheter-related problems. Clin. Nutr..

[65] Pironi L., Cuerda C., Jeppesen P.B., Joly F., Jonkers C., Krznarić Ž., Lal S., Lamprecht G., Lichota M., Mundi M.S. (2023). ESPEN guideline on chronic intestinal failure in adults—Update 2023. Clin. Nutr..

[66] 66. Asgary M.R., Mirghazanfari S.M., Hazrati E., Hadi V., Mehri Ardestani M., Bani Yaghoobi F., Hadi S. (2023). The Effect of L-Citrulline Supplementation on Outcomes of Critically Ill Patients under Mechanical Ventilation; a Double-Blind Randomized Controlled Trial. Arch. Acad. Emerg. Med..

[67] Calder P.C. (2019). Intravenous Lipid Emulsions to Deliver Bioactive Omega-3 Fatty Acids for Improved Patient Outcomes. Mar. Drugs.

[68] Hill A., Elke G., Weimann A. (2021). Nutrition in the Intensive Care Unit—A Narrative Review. Nutrients.

[69] Pradelli L., Mayer K., Klek S., Omar Alsaleh A.J., Clark R.A.C., Rosenthal M.D., Heller A.R., Muscaritoli M. (2020). ω-3 Fatty-Acid Enriched Parenteral Nutrition in Hospitalized Patients: Systematic Review with Meta-Analysis and Trial Sequential Analysis. J. Parenter. Enter. Nutr..

[70] Boullata J.I., Mirtallo J.M., Sacks G.S., Salman G., Gura K., Canada T., Maguire A., ASPEN Parenteral Nutrition Safety Committee (2022). Parenteral nutrition compatibility and stability: A comprehensive review. JPEN J. Parenter Enteral. Nutr..

[71] Osland E.J., Polichronis K., Madkour R., Watt A., Blake C. (2022). Micronutrient deficiency risk in long-term enterally fed patients: A systematic review. Clin. Nutr. ESPEN.

[72] Gudivada K.K., Kumar A., Shariff M., Sampath S., Varma M.M., Sivakoti S., Krishna B. (2020). Antioxidant micronutrient supplementation in critically ill adults: A systematic review with meta-analysis and trial sequential analysis. Clin. Nutr..

[73] Johnsen J.C., Reese S.A., Mackay M., Anderson C.R., Jackson D., Paul I.L. (2017). Assessing Selenium, Manganese, and Iodine Status in Pediatric Patients Receiving Parenteral Nutrition. Nutr. Clin. Pract..

[74] Cameron L.K., Lumlertgul N., Bear D.E., Cooney E., McKenzie C., Ostermann M. (2023). Micronutrient use in critical care: Survey of clinical practice. Clin. Nutr. ESPEN.

[75] Berger M.M., Shenkin A., Schweinlin A., Amrein K., Augsburger M., Biesalski H.K., Bischoff S.C., Casaer M.P., Gundogan K., Lepp H.L. (2022). ESPEN micronutrient guideline. Clin. Nutr..

[76] Hargreaves I.P., Mantle D. (2019). Supplementation with selenium and coenzyme Q10 in critically ill patients. Br. J. Hosp. Med..

[77] Li C., Lu F., Chen J., Ma J., Xu N. (2022). Probiotic Supplementation Prevents the Development of Ventilator-Associated Pneumonia for Mechanically Ventilated ICU Patients: A Systematic Review and Network Meta-analysis of Randomized Controlled Trials. Front. Nutr..

[78] Camblor-Álvarez M., Ocón-Bretón M.J., Luengo-Pérez L.M., Viruzuela J.A., Sendrós-Maroño M.J., Cervera-Peris M., Grande E., Álvarez-Hernández J., Jiménez-Fonseca P. (2018). Soporte nutricional y nutrición parenteral en el paciente oncológico: Informe de consenso de un grupo de expertos Nutritional support and parenteral nutritio [Nutritional support and parenteral nutrition in the oncological patient: An expert group consensus report]. Nutr. Hosp..

[79] Guiducci S., Duci M., Moschino L., Meneghelli M., Fascetti Leon F., Bonadies L., Cavicchiolo M.E., Verlato G. (2022). Providing the Best Parenteral Nutrition before and after Surgery for NEC: Macro and Micronutrients Intakes. Nutrients.

[80] Hill A., Starchl C., Dresen E., Stoppe C., Amrein K. (2023). An update of the effects of vitamins D and C in critical illness. Front. Med..

[81] Newsholme P., Diniz V.L.S., Dodd G.T., Cruzat V. (2023). Glutamine metabolism and optimal immune and CNS function. Proc. Nutr. Soc..

[82] Barnish M., Sheikh M., Scholey A. (2023). Nutrient Therapy for the Improvement of Fatigue Symptoms. Nutrients.

[83] Gunst J., Casaer M.P., Preiser J.C., Reignier J., Van den Berghe G. (2023). Toward nutrition improving outcome of critically ill patients: How to interpret recent feeding RCTs?. Crit. Care.

[84] Berger M.M., Burgos R., Casaer M.P., De Robertis E., Delgado J.C.L., Fraipont V., Gonçalves-Pereira J., Pichard C., Stoppe C. (2022). Clinical nutrition issues in 2022: What is missing to trust supplemental parenteral nutrition (SPN) in ICU patients?. Crit. Care.

[85] Elremaly W., Mohamed I., Rouleau T., Lavoie J.C. (2016). Impact of glutathione supplementation of parenteral nutrition on hepatic methionine adenosyltransferase activity. Redox Biol..

[86] Allen L.V. (2020). Sterile Basics: Intravenous Admixture Preparation Considerations, Part 5: pH Considerations. Int. J. Pharm. Compd..

[87] Zhao B., Gao R., Jiao L., Zhang F., Wang B., Mei D. (2021). Physical Stability of Medium-Chain Triglyceride/Long-Chain Triglyceride Emulsion Injections from 5 Manufacturers in High-Concentration Electrolyte-Based Total Nutrient Admixtures. J. Parenter. Enter. Nutr..

[88] Farhan M., McCallion N., Bennett J., Cram A., O’Brien F. (2023). Stability and compatibility of parenteral nutrition solutions; a review of influencing factors. Eur. J. Pharm. Biopharm..

[89] Peterson S., Dobak S., Phillips W., Malone A., Ireton-Jones C., Haney A., Rybicki M., Guenter P. (2020). Enteral and Parenteral Order Writing Survey-A Collaborative Evaluation Between the Academy of Nutrition and Dietetics’ Dietitians in Nutrition Support Dietetics Practice Group and the American Society for Parenteral and Enteral Nutrition (ASPEN) Dietetics Practice Section. J. Acad. Nutr. Diet..

[90] Lambell K.J., Tatucu-Babet O.A., Chapple L.A., Gantner D., Ridley E.J. (2020). Nutrition therapy in critical illness: A review of the literature for clinicians. Crit. Care.

[91] Blaauw R., Osland E., Sriram K., Ali A., Allard J.P., Ball P., Chan L., Jurewitsch B., Coughlin K.L., Manzanares W. (2019). Parenteral provision of micronutrients to adult patients: An expert consensus paper. J. Parenter. Enter. Nutr..

[92] Unger N., Holzgrabe U. (2018). Stability and assessment of amino acids in parenteral nutrition solutions. J. Pharm. Biomed. Anal..

[93] Gallagher V., Berlana D., Paulsson M., White R.J. (2021). Parenteral nutrition: A call to action for harmonization of policies to increase patient safety. Eur. J. Clin. Nutr..

[94] Baiu I., Spain D.A. (2019). Parenteral Nutrition. JAMA.

[95] Otero-Millán L., Lago Rivero N., Blanco Rodicio A., García Beloso N., Legido Soto J.L., Piñeiro-Corrales G. (2021). Stability of lipid emulsion in total parenteral nutrition: An overview of literature. Clin. Nutr. ESPEN.

[96] Mundi M.S., Nystrom E.M., Hurley D.L., McMahon M.M. (2017). Management of Parenteral Nutrition in Hospitalized Adult Patients [Formula: See text]. J. Parenter. Enter. Nutr..

[97] Arrieta Loitegui M., Gomis Muñoz P., Rosas C., Delmiro Magdalena A., Ferrari Piquero J.M. (2019). Precipitado negro en nutrición parenteral [Black precipitate in parenteral nutrition]. Nutr. Hosp..

[98] Haines K., Grisel B., Gorenshtein L., Wischmeyer P.E. (2023). Lipid emulsions in parenteral nutrition: Does it matter?. Curr. Opin. Crit. Care.

[99] Calder P.C., Waitzberg D.L., Klek S., Martindale R.G. (2020). Lipids in Parenteral Nutrition: Biological Aspects. JPEN J. Parenter Enteral. Nutr..

[100] Novak A., Gutiérrez-Zamora M., Pérez-Lozano P., Suñé-Negre J.M., Llop J.M., Ticó J.R., Miñarro M., García-Montoya E., Van Schepdael A. (2020). Study of tocopherol content and its potential antioxidant activity in commercial lipid emulsions for parenteral nutrition. Pharmazie.

[101] Koletzko B. (2022). 3.5 Parenteral Nutrition Support. World Rev. Nutr. Diet..

[102] Curtis C. (2018). Technology in Parenteral Nutrition Compounding. Nutr. Clin. Pract..

[103] Allen L.V. (2021). Basics of Sterile Compounding: Preparation Considerations, Part 10: Packaging and Container-closure Issues. Int. J. Pharm. Compd..

[104] Mitrus O., Żuraw M., Losada-Barreiro S., Bravo-Díaz C., Paiva-Martins F. (2019). Targeting Antioxidants to Interfaces: Control of the Oxidative Stability of Lipid-Based Emulsions. J. Agric. Food Chem..

[105] Berlana D., Almendral M.A., Abad M.R., Fernández A., Torralba A., Cervera-Peris M., Piñeiro G., Romero-Jiménez R., Vázquez A., Ramírez E. (2019). Cost, Time, and Error Assessment during Preparation of Parenteral Nutrition: Multichamber Bags versus Hospital-Compounded Bags. J. Parenter. Enter. Nutr..

